# Supplementary material for: Off-Target Deletion of Conditional Dbc1 Allele in the Foxp3YFP-Cre Mouse Line under Specific Setting
Source: Cells. 2019 Oct 24;8(11):1309. doi: 10.3390/cells8111309 (PMC6912351; doi:10.3390/cells8111309)
Supplement: Supplementary file 1 [file cells-08-01309-s001.pdf]

Supplementary Figures

Figure S1.

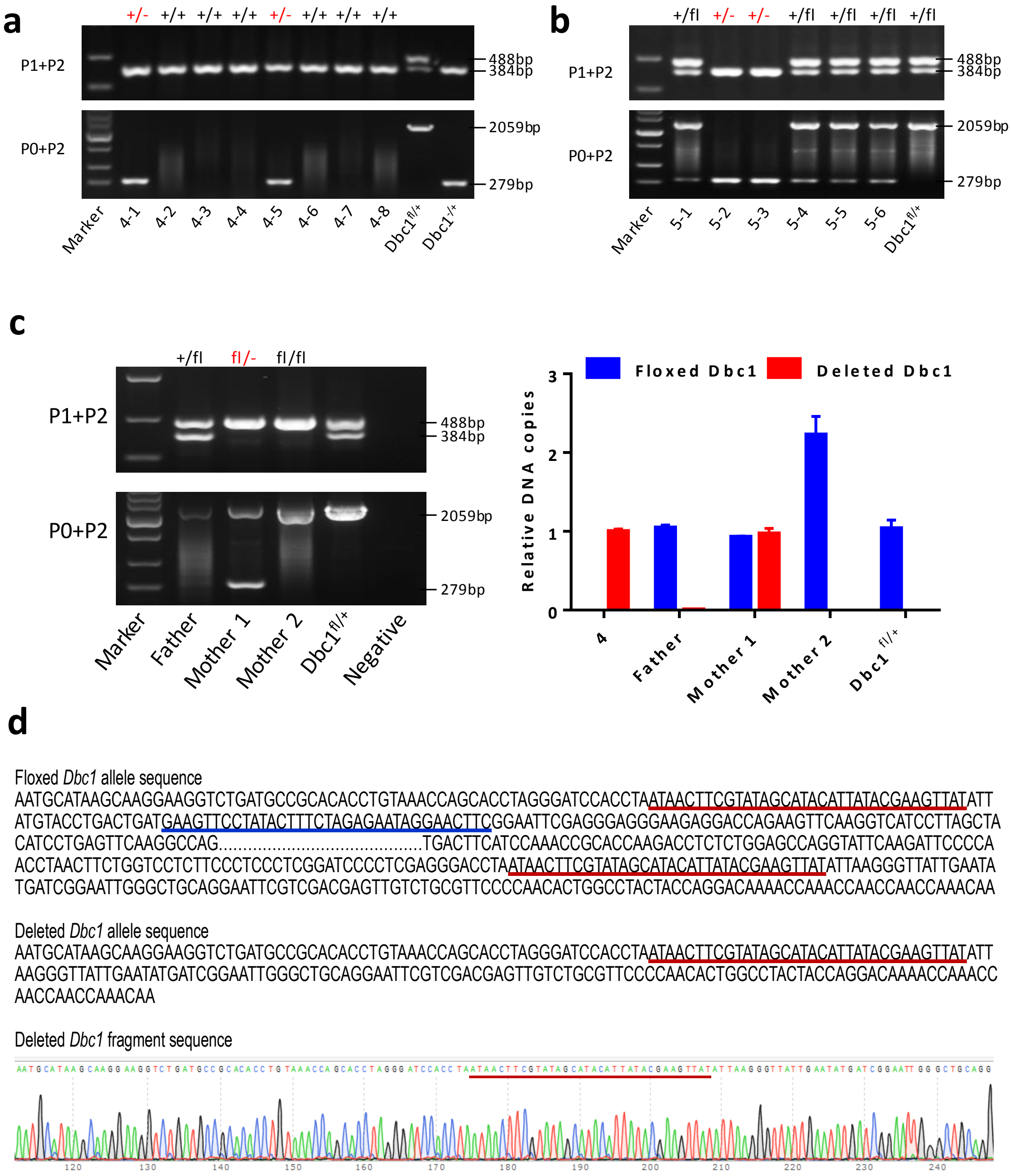

Figure S1. The conditional *Dbc1* allele was completely deleted in some offspring of *Foxp3*$^{Cre/Y}$ *Dbc1*$^{fl/+}$ crossing *Foxp3*$^{Cre/Cre}$ *Dbc1*$^{fl/fl}$ mice. (a) The PCR product sizes of the offspring of mouse #4 in Figure 2a crossing Wt mice using the primer indicated. (b) The PCR product sizes of the offspring of mouse #5 in Figure 2a crossing Wt mice using the primer indicated. (c) Left panel: the PCR product sizes of the father and mother of mouse #4 and #5 in Figure 2a; right panel: the relative amounts of the floxed *Dbc1* and recombined *Dbc1* of father and mother of mouse #4 and #5 in Figure 2a were tested by qPCR. For qPCR, the results were normalized to the DNA sample from *Dbc1*$^{fl/+}$ mice or mouse #4 in Figure 2a. The data were represented as the mean ±

standard deviation of three independent experiments. +/-, +/+, +/fl, fl/- and fl/fl show the genotype of *Dbc1* alleles. (d) The sequence of the deleted *Dbc1* allele. The red underlined sequence is Loxp site; the blue underlined sequence is FRT site.

Figure S2.

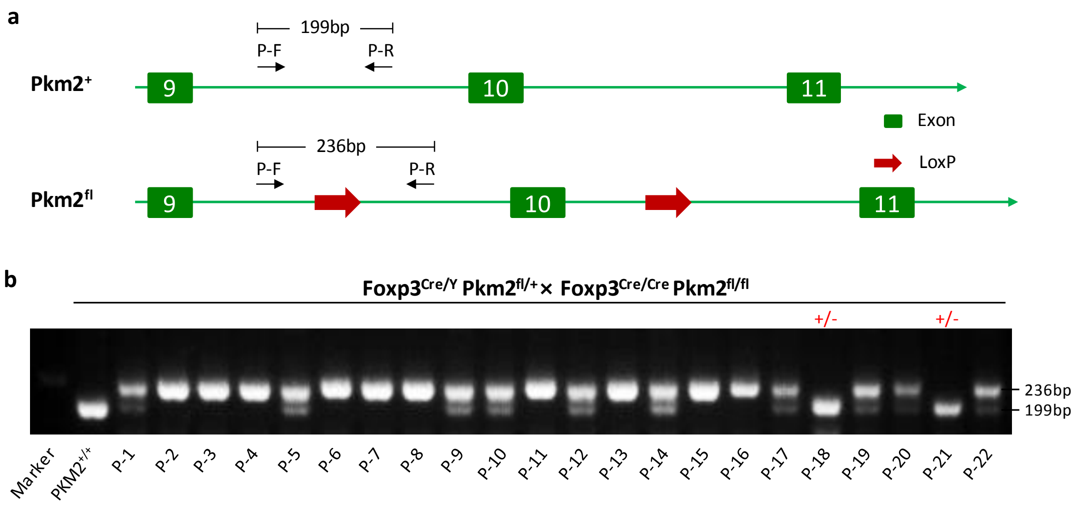

Figure S2. The conditional *Pkm2* allele was completely deleted in some offspring of male *Foxp3*$^{Cre/Y}$ *Pkm2*$^{fl/+}$ mice crossing female *Foxp3*$^{Cre/Cre}$ *Pkm2*$^{fl/fl}$ mice. (a) Scheme of conditional *Pkm2* allele. And the sites of the primer P-F and primer P-R. (b) The PCR product sizes of the offspring of *Foxp3*$^{Cre/Y}$ *Pkm2*$^{fl/+}$ mice crossing female *Foxp3*$^{Cre/Cre}$ *Pkm2*$^{fl/fl}$ mice using the primer indicated. +/- shows the genotype of *Pkm2* alleles.

Figure S3.

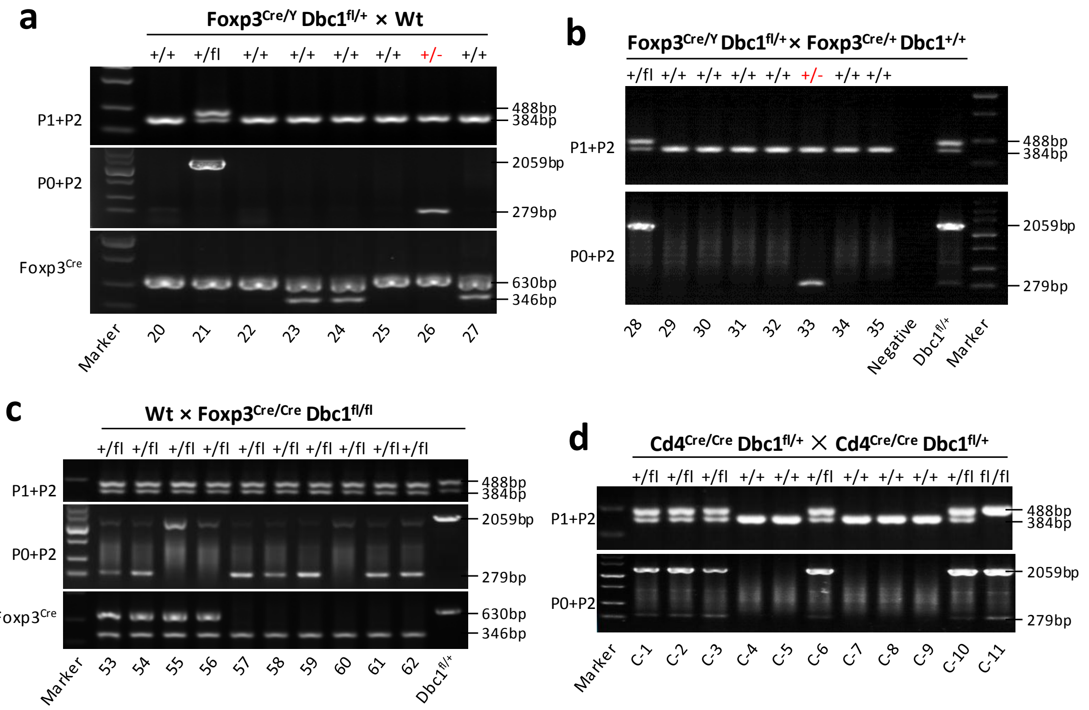

Figure S3. Genotype the mice in the progeny of different breeding pairs. (a) The PCR product sizes of the offspring of *Foxp3*$^{Cre/Y}$ *Dbc1*$^{fl/+}$ × Wt mice using the primer indicated. (b) The PCR product sizes of the offspring of *Foxp3*$^{Cre/Y}$ *Dbc1*$^{fl/+}$× *Foxp3*$^{Cre/+}$ *Dbc1*$^{+/+}$ mice using the primer indicated. (c) The PCR product sizes of the offspring of Wt × *Foxp3*$^{Cre/Cre}$ *Dbc1*$^{fl/fl}$ mice using the primer indicated. (d) The PCR product sizes of the offspring of female *CD4*$^{Cre/Cre}$ *Dbc1*$^{fl/+}$ mice crossing with male *CD4*$^{Cre/Cre}$ *Dbc1*$^{fl/+}$ mice using the primer indicated. +/-, +/+, +/fl, and fl/fl show the genotype of *Dbc1* alleles. The panel of *Foxp3*^{Cre} shows the genotype of *Foxp3*-IRES-YFP-Cre alleles.

Figure S4.

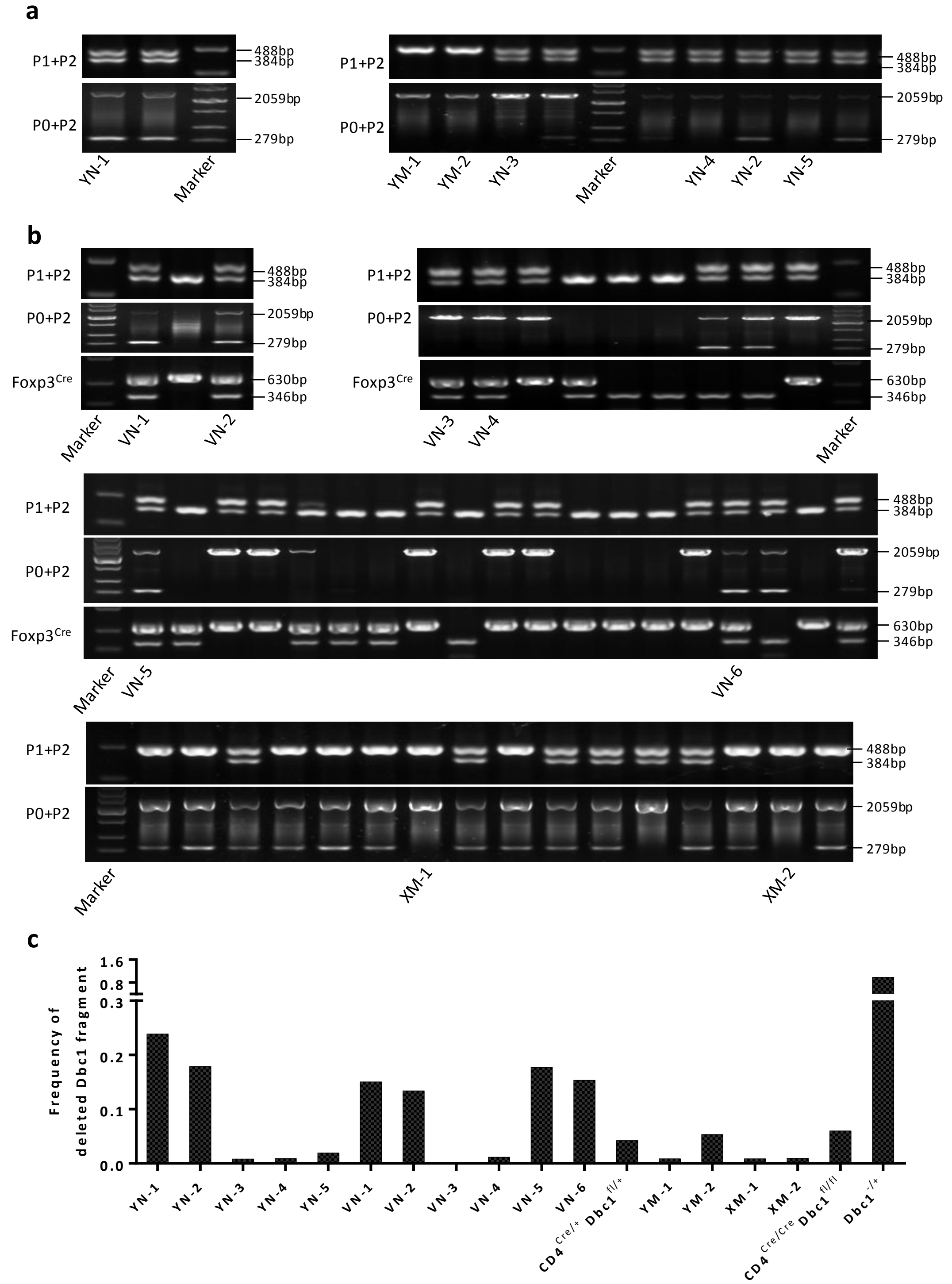

Figure S4. The genotype of mice chosen to breed. (a) The genotype of male mice chosen to breed in table 1. (b) The genotype of female mice chosen to breed in table 1. (c) The frequency of recombined *Dbc1* fragment in the mice chosen to breed in table 1.

Figure S5.

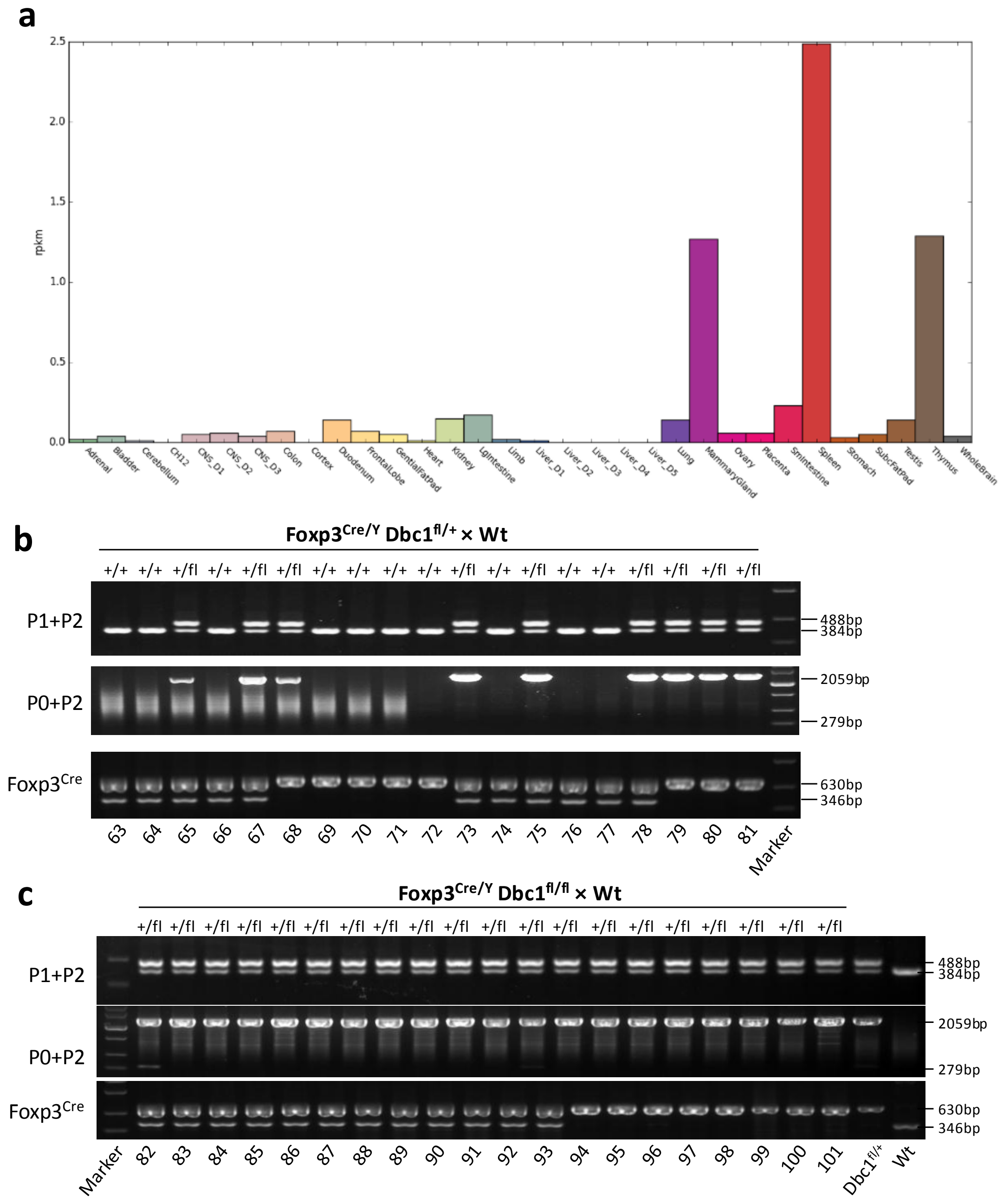

Figure S5. The expression of *Foxp3* in the testis did not cause knockout of *Dbc1* allele in the offspring of *Foxp3*$^{Cre/Y}$ *Dbc1*$^{fl/+}$ mice

(a) *Foxp3* expression across multiple tissues/cell lines (Data come from ENCODE). (b) The PCR product sizes of the offspring of Wt female mice crossing male *Foxp3*$^{Cre/Y}$ *Dbc1*$^{fl/+}$ mice with a low frequency of recombined *Dbc1* fragment using the primer indicated. (c) The PCR product sizes of the offspring of Wt female mice crossing male *Foxp3*$^{Cre/Y}$ *Dbc1*$^{fl/fl}$ mice with a low frequency of recombined *Dbc1* fragment using the primer indicated. +/+, and +/fl show the genotype of *Dbc1* alleles. The panel of *Foxp3*^{Cre} shows the genotype of *Foxp3*-IRES-YFP-Cre alleles.

Figure S6.

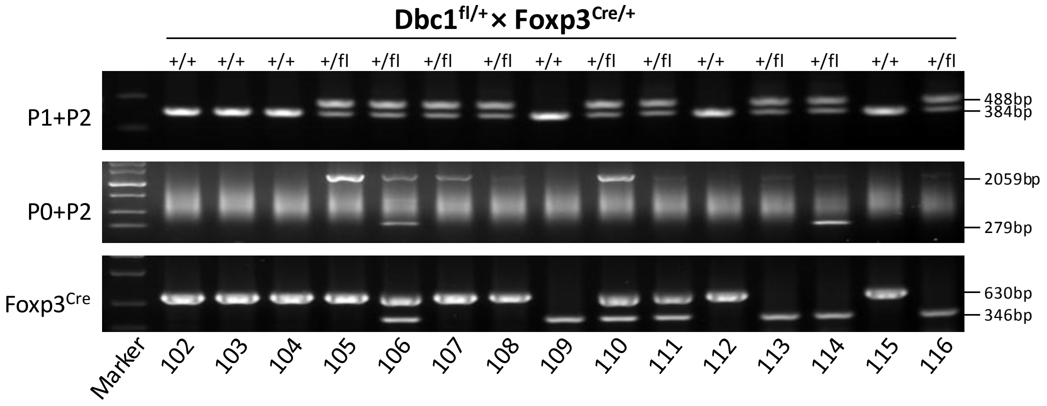

Figure S6. The *Dbc1* alleles of some fetuses in the progeny of *Foxp3*^Cre^ *Dbc1*^fl^ mice were recombined. The PCR product sizes of the offspring of male *Dbc1*$^{fl/+}$ mice crossing female *Foxp3*$^{Cre/+}$ mice using the primer indicated. +/+, and +/fl show the genotype of *Dbc1* alleles. The panel of *Foxp3*^{Cre} shows the genotype of *Foxp3*-IRES-YFP-Cre alleles.
